# The steering gaits of sperm

**DOI:** 10.1098/rstb.2019.0149

**Published:** 2019-12-30

**Authors:** A. Gong, S. Rode, U. B. Kaupp, G. Gompper, J. Elgeti, B. M. Friedrich, L. Alvarez

**Affiliations:** 1 Center of Advanced European Studies and Research (CAESAR), Molecular Sensory Systems, Ludwig-Erhard-Allee 2, 53175 Bonn, Germany; 2 Theoretical Soft Matter and Biophysics, Institute of Complex Systems and Institute for Advanced Simulation, Forschungszentrum Jülich, 52425 Jülich, Germany; 3 Biological Algorithms Group, TU Dresden, Biological Systems Path of the Center for Advancing Electronics Dresden (CFAED), Helmholtzstrasse 18, 01069 Dresden, Germany

**Keywords:** cytoskeleton, symmetry-breaking, navigation, chirality, cilia

## Abstract

Sperm are highly specialized cells, which have been subject to substantial evolutionary pressure. Whereas some sperm features are highly conserved, others have undergone major modifications. Some of these variations are driven by adaptation to mating behaviours or fitness at the organismic level. Others represent alternative solutions to the same task. Sperm must find the egg for fertilization. During this task, sperm rely on long slender appendages termed flagella that serve as sensory antennas, propellers and steering rudders. The beat of the flagellum is periodic. The resulting travelling wave generates the necessary thrust for propulsion in the fluid. Recent studies reveal that, for steering, different species rely on different fundamental features of the beat wave. Here, we discuss some examples of unity and diversity across sperm from different species with a particular emphasis on the steering mechanisms.

This article is part of the Theo Murphy meeting issue ‘Unity and diversity of cilia in locomotion and transport’.

## 1. Introduction

For fertilization, sperm from different species are confronted with different challenges. For example, sperm must penetrate the protecting egg coat before reaching the plasma membrane of the egg. For most mammals, sperm can penetrate this coat at any region, whereas fish and insect sperm must locate and traverse the micropyle, a minuscule and narrow canal on the egg's coat [1]. Finding and penetrating the micropyle can be further complicated for short-lived sperm, as is the case of fish sperm whose motility ceases within a few minutes after activation [2]. In promiscuous internal fertilizers, sperm are confronted with another challenge: they must reach the egg before their competitors. To succeed, these species have evolved fast-swimming sperm [3] or adapted other strategies, such as sperm–sperm collaboration [4].

Also, the rheological properties of the environment along the path to the egg can vary substantially. External fertilizers, such as sea urchins, release sperm and eggs into the seawater (broadcast spawners). By contrast, males from internal fertilizers, such as mammals, deposit sperm in the complex female reproductive tract, where sperm interact with the boundary walls and must progress through the highly viscous mucus that lines the reproductive tract tissue [5]. It has been proposed that to deal with such high viscosities, human sperm, for instance, have developed additional structures that render their flagellum more stable to buckling [6]. Furthermore, progression through mucus, detachment from the epithelia and penetration of egg vestments are supported by hyperactive sperm motility, a transient vigorous motility pattern commonly observed across mammals [7]. Signals used for navigation also differ, and sperm use a highly specialized repertoire of species- and sperm-specific signalling molecules, including chemoattractants, receptors, ion channels and solute carriers [8–10].

**Figure 1.**
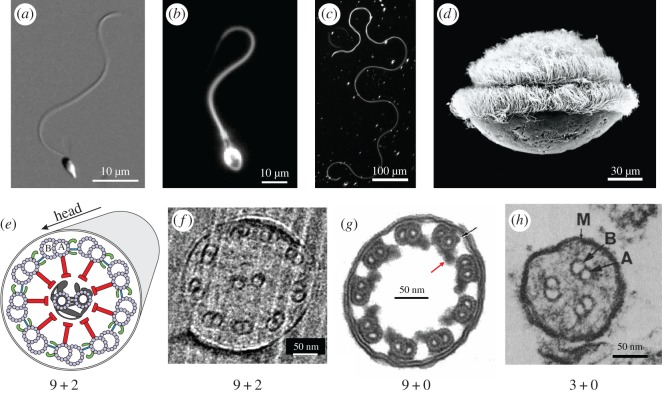
Unity and diversity in sperm morphology and ultrastructure. Exemplary variations in sperm morphology (*a*–*d*) and axonemal structures (*e*–*h*). (*a*) Sperm from the sea urchin *Arbacia punctulata* characterized by the stereotypical ‘primitive’ sperm morphology. (*b*) Human sperm featuring a thicker flagellum with outer dense fibres surrounding the 9 + 2 structure. (*c*) Sperm cell from the fruit fly, *Drosophila melanogaster*, with a 2 mm-long flagellum. (*d*) Multiflagellated sperm from the plant *Cycas revoluta*. Reprinted with permission from [11]. (*e*) Schematic cross-section of the highly conserved 9 + 2 axonemal structure present in most motile cilia and flagella. Nine microtubule doublets (with tubules A and B) are arranged cylindrically around an additional pair of microtubule singlets located at the centre. Two dynein arms (green), referred to as inner and outer dynein arms, contain different subsets of dynein molecular motors. Arms are projected from the A tubule of one microtubule doublet towards the B tubule of the adjacent doublet in a clockwise fashion. (*f*) 9 + 2 axonemal structure of sperm from sea urchin. (*g*) 9 + 0 axonemal structure from sperm of the eel *Anguilla anguilla*. Only the inner dynein arms are visible (red arrow). Instead of outer dynein arms, a small electron-dense structure can be occasionally seen (black arrow). Adapted from [12]. (*h*) The 3 + 0 axoneme from the sperm of the parasite *Diplauxis hatti* is the simplest axoneme known. Adapted from [13]. Panels (*a*,*b*) indicate the two tubules of the microtubule doublets. M indicates the plasma membrane. Axonemal structures (*e*–*h*) are viewed from the head towards the flagellar tip. Dynein motors in (*f*) and (*h*) have not been resolved.

These diverse variations of geometrical and physical constraints during fertilization have resulted in large variations of overall sperm morphology and the underlying microscopic structures (figure 1). Phylogenetic studies suggest that a ‘primitive’ sperm type with a simple morphology appeared early in evolution with the multicellular animals (Metazoa) and that this morphology has been preserved among many widely separated phyla [14,15]. As a general rule, external fertilizers releasing sperm freely into the water still preserve such primitive form and variations from it can be found for species with internal fertilization or where sperm is delivered in some way to the female (for example, via sperm capsules or spermatophores). This ‘primitive’ sperm morphology consists in a coarse sense of a small head (usually about 2–5 µm), and a longer tail (usually about 10–50 µm), a single eukaryotic flagellum. The flagellum contains few (1–5) mitochondria, and the cytoskeletal scaffold, termed the axoneme, to which dynein molecular motors are attached along its entire length [14,16,17]. These sperm by and large are rotationally symmetric with respect to the long axis of the head. Echinoderms, such as sea urchin or starfish, produce sperm featuring the typical ‘primitive’ morphology (figure 1*a*). Mammalian sperm display a similar morphology, yet their flagellum is thicker owing to the outer dense fibres that run parallel to the axoneme as well as a larger number of mitochondria (figure 1*b*). Sperm from the fruit fly, *Drosophila melanogaster*, possess a very long flagellum that is about the size of the animal itself (*ca* 2 mm; figure 1*c*) [18] and display a complex motility pattern (electronic supplementary material, movie S1). Other flies, such as *Drosophila bifurca*, produce gigantic sperm that can be as long as 58 mm [19]. Motile sperm from plants are typically biflagellated [20], and in some species, such as *Cycas revoluta*, motile flagella can be counted by the thousands (figure 1*d*).

The cytoskeletal scaffold inside the flagellum, the axoneme, drives sperm motility. The axoneme is built from nine microtubule doublets running along the length of the sperm flagellum and circularly arranged in juxtaposition to the cell membrane (figure 1*e,f*). From each microtubule doublet, bundles of dynein molecular motors (dynein arms) project towards the adjacent microtubule doublet in a clockwise sense (when viewed from the head towards the flagellar tip). In the centre of this cylindrical arrangement of microtubule doublets, commonly an additional set of two microtubules can be found. The resulting 9 + 2 cytoskeletal structure is highly conserved across sperm and other ciliated cells [21], yet, interesting variations are found in nature (figure 1*f–h*) [8]. In some species, such as the eel *Anguilla anguilla*, the sperm axoneme lacks the central microtubule pair and the corresponding accessory structures (figure 1*g*). Such a structure is known as the 9 + 0 axoneme and can also be found in nodal cilia [22]. Both nodal cilia and eel sperm display a non-planar beat pattern. Eel sperm also lack the outer dynein arm and display very high beat frequencies (about 150 Hz compared with about 10–30 Hz for sperm from mammals or about 50 Hz for sea urchin sperm). Perhaps the simplest axoneme that has been found in nature is that from the parasite protozoan *Diplauxis hatti* [13], which comprises only three microtubule doublets and lacks the central pair (3 + 0 axoneme; figure 1*h*). The reader interested in sperm morphological diversity is referred to [15].

Despite the large variations, motile sperm are confronted with the same task: they must locate the egg for fertilization. For this, sperm have developed the ability to steer using the flagellum. To understand the mechanism and principles underlying steering, it is important to comprehend the self-propulsion of microorganisms at low Reynolds numbers [23]. In the following, we will discuss fundamental requirements that allow a sperm cell to change its swimming direction. We will compare two mechanisms to change direction and discuss the parameter space that can be exploited for steering. Finally, we will illustrate how sperm from different species employ these mechanisms.

This review is intended to provide a basic understanding of sperm steering. Several simplifying assumptions will be made, as follows. We only consider uniflagellated sperm and assume a prescribed flagellar beat pattern that is planar and perfectly periodic in time. The reader interested in steering mechanisms and swimming gaits of multiflagellated cells is referred to the work of Wan [24]. For pedagogical reasons, we will employ a simplified hydrodynamic theory of flagellar self-propulsion known as resistive-force theory [25], which neglects hydrodynamic interactions between different parts of the sperm cell or nearby boundary surfaces. Readers interested in hydrodynamic subtleties involved in self-propulsion of sperm cells are referred to [26–28].

## 2. Low Reynolds numbers imply instantaneous terminal velocity

Is water viscous? The answer to this question depends on the relevant length-scale (*L*) and velocities (*v*) of the body that moves in this fluid. Specifically, given a value of about *η* = 10^−3^ Pa s for the dynamic water viscosity, it is not possible to affirm whether this value is small or large. We can only compare this value with other values and make a statement based on a dimensionless ratio.

The dimensionless Reynolds number ratio serves such comparison: *Re* = *ρLv*/*η*. It characterizes the importance of inertial forces relative to viscous forces. For a macroscopic object moving in water (fluid density *ρ* = 10^3^ kg m^−3^), such as a swimming human or a propelling boat, this number will be large, yet for a microswimmer such as a sperm cell with characteristic length-scale of about *L* = 50 µm and a characteristic flagellar wave velocity *v* = *λ*/*T* ≈ 1 mm s^−1^, the Reynolds number will be small. What are the physical implications for self-propulsion of a small Reynolds number?

It is useful to start with a macroscopic example close to our everyday perception. When an object initially at rest starts to sink in a fluid under the influence of the gravitational force, the object will first accelerate. As the speed increases, the drag forces exerted by the fluid on the object increase until a balance between gravitational, buoyancy and drag forces is established. Then, the net force acting on the object becomes zero, and the object moves at a constant terminal velocity. Sperm propel themselves in a liquid by undulating their flagellum. While moving, the flagellum pushes the fluid, and the opposing reaction force acts on the cell, giving rise to a thrust force. If we restrained the sperm cell from moving, e.g. by holding the sperm head with a micropipette, we would have to apply a constraining force that is equal in magnitude and opposite to this thrust force. For a freely swimming sperm cell, however, this thrust force is counterbalanced by friction forces resulting from fluid drag.

The Reynolds number (ratio between inertia and viscous forces) for sperm swimming in a watery fluid is very small and, accordingly, inertia is negligible. As a consequence, the time to reach terminal velocity is also negligible, and active forces exerted by moving parts of the flagellum are always perfectly balanced by local hydrodynamic drag forces. A similar argument shows that not only do forces balance, but also does the net torque about any pivot, and, as a result, linear and angular velocities reach terminal velocities almost instantaneously.

Sperm density is similar, yet not identical, to that of the surrounding fluid. Owing to a small density mismatch, sperm suspensions tend to be inhomogeneous. If allowed to settle, a sperm density gradient will form in time, and higher sperm densities will become apparent at the bottom of the suspension. Such effects can be relevant in some instances, such as that of bioconvection of microorganisms [29]. However, for most purposes, swimming forces dominate over buoyancy or gravitational forces. We will thus assume that sperm is neutrally buoyant and neglect the effect of gravitational forces. In the absence of further external forces, a sperm cell swimming at low Reynolds number can be considered force-free and torque-free. The condition of force-free and torque-free swimming allows computation of the translational and rotational velocities of an undulatory swimmer with a prescribed beat pattern in a self-consistent manner (electronic supplementary material, note S1). In the following, we will characterize the flagellar beat pattern by the flagellar curvature *C*(*s,t*), where *s* is the arc-length coordinate along the flagellum and *t* the time, and we will compute the motion of sperm using the resistive-force theory established by Gray & Hancock [25]. As a note, this theory shows that sperm propulsion is enabled by the fact that a slender object has unequal friction coefficients *ξ*_||_ and *ξ*_⊥_ for tangential and perpendicular motions, respectively. For a thin rod, the ratio *ξ*_R_ = *ξ*_⊥/_*ξ*_||_ is approximately 2. The anisotropy ratio can be selected to fit data for different species, waveforms and surrounding environment. Previous studies on bull sperm found that a value of *ξ*_R_ ≈ 1.81 produced an excellent fit to the experimental data [30]. In the following, we will use this value for quantitative estimates of swimming parameters.

## 3. Changing direction requires beat patterns with broken symmetry

From a morphological perspective, the simple uniflagellated sperm cell is symmetric along the long cell axis. Hence, propulsion by a symmetric bending wave would result in straight swimming. This can be easily understood by symmetry arguments, but a formal mathematical description can be useful. For a sperm cell propelled by a flagellar beat characterized by its flagellar curvature, any beat pattern by which the flagellum becomes its mirror image after half a beat period *T*/2:

3.1 $$C(s,\;t)=-C\left(s,\;t+\frac{T}{2}\right),$$

will result in straight swimming because the average cell rotation attained during the first half of the beat period will be cancelled during the second half. Since the beat pattern and hence its curvature *C*(*s, t*) is assumed to be periodic in time, we can write down its expansion into Fourier modes. To highlight the functional implications of the symmetry condition embodied in equation (3.1), we write such an expansion for both the curvature function *C*(*s*, *t*) and the half-a-period delayed function *C*(*s*, *t* + *T*/2):

3.2 $$\begin{array}{r} C(s,\;t)=c_{0}(s)+\sum_{n=1}^{\infty }c_{n}(s)\cos\left(\frac{2\pi n}{T}t\right)+{\bar{c}}_{n}(s)\sin\left(\frac{2\pi n}{T}t\right) \end{array}$$

and

3.3 $$\begin{array}{rl} C & \left(s,\;t+\frac{T}{2}\right)=\\ & c_{0}(s)+\sum_{n=1}^{\infty }{\left(-1\right)}^{n}\left[c_{n}(s)\cos\left(\frac{2\pi n}{T}t\right)+{\bar{c}}_{n}(s)\sin\left(\frac{2\pi n}{T}t\right)\right]. \end{array}$$

By substituting equations (3.2) and (3.3) in equation (3.1), we find that for a symmetric beat pattern the coefficients of all even (*n* = 2*j* with *j* positive integer or zero) Fourier modes must vanish. Thus, to take a turn, the flagellar beat must comprise a non-zero average curvature (*n* = 0) or higher curvature modes that are even (*n* = 2, 4, … , 2*j*). In the following, we will address three prototypical cases: (a) a flagellar beat pattern whose curvature profile is a sinusoidal travelling wave with amplitude *C*_1_, (b) a superposition of a sinusoidal wave and an average curvature component *C*_0_, and (c) a superposition of a sinusoidal wave and a second harmonic component with amplitude *C*_2_:

3.4a $$C(s,\;t)=C_{1}\sin(ks-\omega_{0}t),$$

3.4b $$C(s,\;t)=C_{0}+C_{1}\sin(ks-\omega_{0}t)$$

and

3.4c $$C(s,\;t)=C_{1}\sin(ks-\omega_{0}t)+C_{2}\sin(ks-2\omega_{0}t+\phi ).$$

Here, *ϕ* denotes a phase shift between first and second beat harmonics, *k* = 2*π*/*λ* is the wavenumber, and *ω*_0_ = 2*π*/*T* the angular beat frequency. For the sake of simplicity, we assume that the wavelength *λ* equals the flagellar length (*λ* = *L*).

As a note, equations (3.4*a*–*c*) can be written in a form more akin to that used in equation (3.2) by defining:

$$\begin{array}{rl} & c_{0}(s)=C_{0};\\ & c_{1}(s)=C_{1}\sin(ks);\\ & {\bar{c}}_{1}(s)=-C_{1}\cos(ks);\\ & c_{2}(s)=C_{2}\sin(ks+\phi );\\ & {\bar{c}}_{2}(s)=-C_{2}\cos(ks+\phi ). \end{array}$$

In the following, we will use the simpler form shown in equations (3.4*a*–*c*).

**Figure 2.**
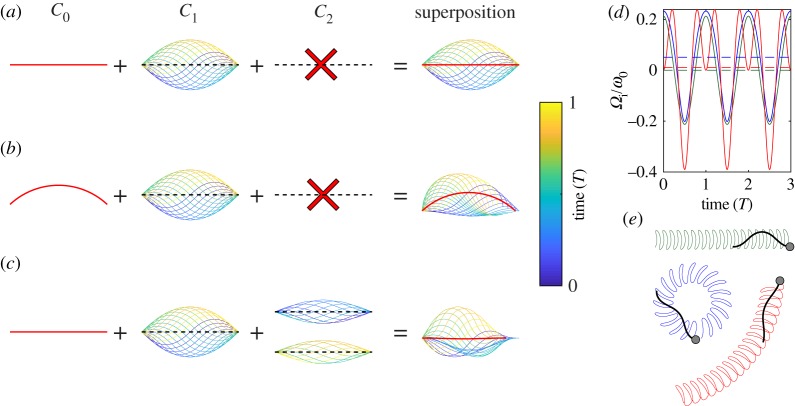
Chiral flagellar beat patterns result in net cell rotation. (*a*–*c*) Prototypical flagellar beat patterns corresponding to (*a*) a symmetric travelling wave with amplitude *C*_1_, (*b*) a superposition of a symmetric travelling wave and a circular arc with constant curvature *C*_0_, and (*c*) a superposition of a symmetric travelling wave and a second harmonic component with amplitude *C*_2_. The two half periods of the second harmonic component, otherwise overlapping, have been shifted vertically for better visualization. The superposition of the average curvature *C*_0_ and the travelling wave (*b*) results in an asymmetric beat in space that can be used as a rudder to make a turn. The beat pattern in (*c*) is characterized by a symmetric average shape (red line), but nonetheless breaks symmetry as it is reflected by the asymmetric envelope of the resultant beat pattern. This beat pattern can also be used to make a turn. (*d*,*e*) Computed instantaneous rotation velocity *Ω*_i_ normalized by the angular beat frequency during three consecutive beat cycles (*d*) and swimming paths (*e*) for sperm featuring a simple travelling wave (olive), or a travelling wave plus an average curvature (blue) or a second harmonic (red). Numerical computations used resistive-force theory and headless sperm models for simplicity. Dashed lines in (*d*) represent the time-average. Parameters: flagellar length *L* = 40 µm, *C*_0_ = −1.5/*L*, *C*_1_ = 0.75*k*, *C*_2_ = *C*_1_/2, *ϕ* = *π*/2, *k* = 2*π*/*λ* and *λ* = *L*.

Equation (3.4*b*) corresponds to an arched beat waveform and is asymmetric in space. Equation (3.4*c*) corresponds to a flagellar beat that is accelerated (decelerated) when both harmonic components have the same (opposite) sign, and despite the fact that the individual dynamic modes are symmetric, their superposition is not (figure 2). This occurs because the two modes have different periods (*T*_1_ = *T* and *T*_2_ = *T*/2). So while individual modes reverse sign under the time-shift *t* ≥ *t* + *T*_1,2_/2 for their respective periods *T*_1_, *T*_2_ (see equation (3.1)), the sum of both terms does not. Equation (3.4*c*) can be rewritten as a single sinusoidal travelling wave with a time-dependent amplitude and phase (see electronic supplementary material, note S2). The time-dependent amplitude results in an asymmetric envelope. The beat patterns described by equations (3.4*b* and 3.4*c*) break the symmetry condition equation (3.1), and result in a non-zero net rotation velocity of sperm (figure 2*d*,*e*).

In general, the coefficients *C*_0_, *C*_1_ and *C*_2_ might be functions of the arc-length coordinate *s*. In the following, we neglect any such spatial dependency and assume these terms to be constant along the arc-length. The interested reader is referred to the literature for specific arc-length dependencies on *C*_1_ and *C*_0_ [30–32] and to the electronic supplementary material, note S3 to facilitate the comparison across studies.

## 4. The steering parameter space

To find the egg, sperm capture sensory cues from their surroundings and transduce this sensory information into steering responses using cellular pathways that ultimately modulate the shape of the flagellar beat [33]. Which flagellar beat parameters can be exploited by the cell for navigation?

We calculated the net rotational velocity *Ω*(*t*) for sperm cells whose flagellar beat pattern features an average flagellar curvature *C*_0_ (equation (3.4*b*)) or a second harmonic with amplitude *C*_2_ (equation (3.4*c*)). Using resistive-force theory and assuming beat patterns with a small-curvature (electronic supplementary material, note S1), we find

4.1 $$\frac{\Omega }{\omega_{0}}=A_{0}k^{-3}C_{1}^{2}C_{0}$$

and

4.2 $$\frac{\Omega }{\omega_{0}}=A_{2}k^{-3}C_{1}^{2}C_{2}\sin\phi ,$$

with proportionality factors *A*_0_(*ξ*_R_) and *A*_2_(*ξ*_R_) (for a more detailed account of these factors, see electronic supplementary material, note S1).

Equations (4.1) and (4.2) reiterate that, for changing direction, sperm require either a non-zero mean curvature *C*_0_ or a second harmonic with non-zero amplitude *C*_2_. Interestingly, in the latter case, turning is only possible if the phase shift *ϕ* between the first and the second harmonic mode is different from 0 and *π*. In both cases, the rotational velocity is proportional to the square of the amplitude *C*_1_ of the fundamental travelling wave. An analogous scaling relation has been found for the net translational velocity [34,35] and has been referred to as the quadratic law of propulsion at low Reynolds number [36]. We will refer to *C*_2_ sin *ϕ* as the effective strength of the second harmonic. From the numerical values of the proportionality factors *A*_0_ and *A*_2_ (*A*_0_ ≈ −0.36; *A*_2_ ≈ −0.04), we find that an average curvature generates an about 10-fold faster rotation than a second harmonic of similar value.

**Figure 3.**
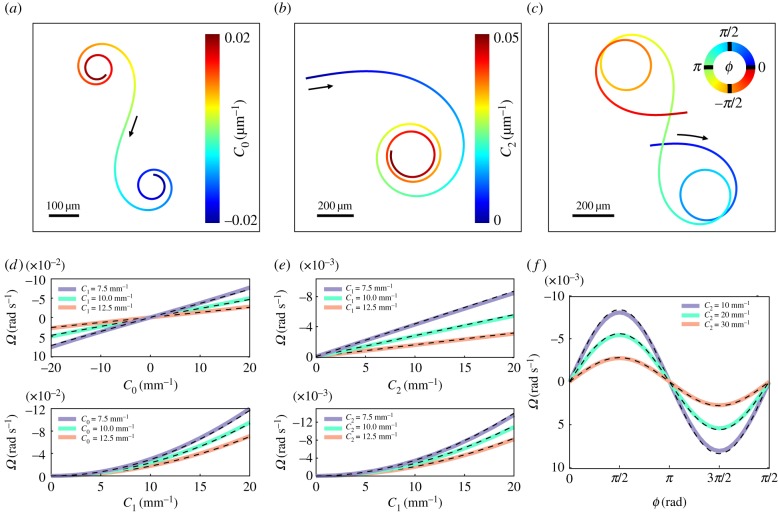
Parameter space of flagellar beat patterns relevant for changing direction. (*a*) Swimming path for the prototypical flagellar waveform of equation (3.4*b*) for a time-dependent mean curvature *C*_0_(*t*). *C*_0_ is varied in time as indicated by the colour code, whereas *C*_1_ = 0.05 µm^−1^ is kept constant. (*b*) Swimming paths for the flagellar waveform of equation (3.4*c*) with time-dependent amplitude *C*_2_(*t*) of the second harmonic, constant *C*_1_ = 0.1 µm^−1^ and second harmonic phase shift *ϕ* = *π*/2. (*c*) Analogous to panel (*b*), but for constant *C*_2_ and a time-varying phase *ϕ*(*t*). Black arrows indicate swimming direction. (*d*–*f*) Net rotation velocity for different (static) parameter values of prototypical flagellar beat patterns, as in equation (3.4*b*) (*d*) or equation (3.4*c*) (*e*,*f*). Solid lines represent numerical computations using the resistive-force theory for a headless sperm cell. Dashed lines represent the approximate analytical solutions calculated from the small-curvature approximation (equations (4.1) or (4.2)). Parameters: *ϕ* = *π/*2 in (*e*) and *C*_1_ = 0.05 µm^−1^ in (*f*).

We compared equations (4.1) and (4.2), which are strictly valid only in the limit of small beat amplitudes, with numerical computations and found good agreement even for realistic parameter values (figure 3). Next, we investigated the effect of a beat pattern whose parameters gradually change as a function of time (figure 3*a*–*c*): By increasing the absolute value of the mean flagellar curvature *C*_0_, we obtain a swimming path characterized by a monotonically increasing absolute value of the path curvature. For *C*_0_ = 0, the swimming path is straight, whereas for *C*_0_ > 0 (*C*_0_ < 0) the swimming velocity rotates in the clockwise (counterclockwise) direction (figure 3*a*). Consistent with equation (4.1), the rotational velocity is linear with the average curvature (figure 3*d*). Similarly, by increasing the amplitude of the second harmonic, also the absolute value of the swimming path curvature increased (figure 3*b*). As predicted by equation (4.2), the rotational velocity depends linearly on the amplitude *C*_2_ of the second harmonic *C*_2_, yet with a slope that is reduced compared with the case of non-zero average curvature *C*_0_ (figure 3*e*). Finally, variations of the phase shift *ϕ* between the first and the second harmonic also resulted in changes of the path curvature (figure 3*c*), and the dependence of the rotational velocity on sin*ϕ* predicted by equation (4.2) was reproduced by numeric computations (figure 3*f*).

## 5. Sperm use the average curvature and the second harmonic for steering

These insights show that morphologically symmetric sperm with a planar flagellar beat can change their swimming direction by using an average curvature or higher even (*n* = 2*j*) harmonics of the beat, but are these turning mechanisms used in nature?

The first mechanism, a non-zero average curvature, has been demonstrated for sperm from numerous species including sea urchin, tunicates, bull, ram and mouse. Specifically, it has been shown that the flagellar beat is composed of a travelling wave superimposed on to an arc of approximately constant curvature [30,31,37,38]. Furthermore, for some species, it has been shown that the rotation velocity depends indeed linearly on *C*_0_ [30,39,40], as predicted by theory. Note that the terminology and the parameters used to characterize the flagellar beat pattern have varied substantially over the last half a century, making a direct comparison across studies difficult. For the convenience of the reader, we include a summary of the different flagellar beat representations in the electronic supplementary material, note S3.

**Figure 4.**
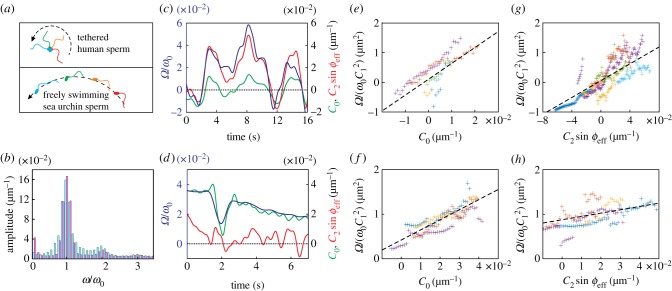
The average flagellar curvature and the second harmonic of the beat are used in nature to change swimming direction. (*a*) Schematic of the experimental conditions used to record the flagellar beat of human and sea urchin sperm. Sea urchin sperm were freely moving, whereas human sperm were tethered at the head to prevent cell rolling and facilitate flagellar tracking. Tethering resulted in a rotation around the head. (*b*) Average power spectrum of the flagellar curvature for human sperm (blue; average from *n* = 6 cells) and sea urchin sperm (pink; average from *n* = 4 cells). (*c*) Exemplary time course of the normalized rotational velocity (blue) of human sperm, as well as the time course of the average flagellar curvature (green) and the effective strength of the second harmonic (red). (*d*) The same as (*c*), but for sea urchin sperm. (*e*,*f*) Normalized rotational velocity versus the average curvature for human (*e*) and sea urchin sperm (*f*). (*g*,*h*) Normalized rotational velocity versus the effective strength of the second harmonic for human (*g*) and sea urchin sperm (*h*). Different colours in *e*–*h* represent different cells. Black dashed line corresponds to a global linear fit. The phase *ϕ*_eff_ is chosen such as to maximize the correlation coefficient between the rotational velocity and the second harmonic strength.

Only recently [41], it has been recognized that steering can also be achieved by using other beat harmonics. In these experiments, human sperm were studied using fast video microscopy. The head of individual sperm cells was tethered to the wall of an observation chamber, which facilitated tracking of the beating flagellum while the cells were still free to rotate around the tethering point (figure 4*a*). Thus, the different beat components and the rotational velocity around the tethering point could be quantified.

In this study, using the same computational tools, we analysed the flagellar beat of freely swimming sea urchin sperm and that from tethered human sperm (figure 4*a*). While equations (4.1) and (4.2) predict the rotational velocity for freely swimming sperm, a slight modification applies for tethered sperm:

5.1 $$\frac{\Omega }{\omega_{0}}=B_{0}k^{-3}C_{1}^{2}C_{0}$$

and

5.2 $$\frac{\Omega }{\omega_{0}}=B_{2}k^{-3}C_{1}^{2}C_{2}\sin(\phi +0.56\pi ),$$

with proportionality factors *B*_0_(*ξ*_R_) and *B*_2_(*ξ*_R_) (see electronic supplementary material, note S1). An estimate of the proportionality factors yields *B*_0_ ≈ −0.102 and *B*_2_ ≈ 0.081. Hence, when a sperm cell is tethered at its head, an average curvature *C*_0_ and a second harmonic with amplitude *C*_2_ contribute about equally to the rotational velocity.

A comparison of the power spectrum of the flagellar curvature reveals that the average curvature *C*_0_ is larger for sea urchin sperm as compared with human sperm, while the second harmonic is somewhat larger for human sperm (figure 4*b*–*d*).

Although we cannot directly manipulate *C*_0_ and *C*_2_ in experiments, we took advantage of the fact that these parameters changed as a function of time, which caused substantial variations in the net rotational velocity (figure 4*c*,*d*). We find that these time-varying values of the rotational velocity correlate with the time-varying values of *C*_0_ and *C*_2_. This correlation is consistent with a linear dependence of the rotational velocity on both average curvature and the effective strength of the second harmonic (figure 4*e*–*h*), in agreement with equations (4.1), (4.2), (5.1) and (5.2).

## 6. Discussion

Symmetry-breaking occurs in biology at every level. From the choice of specific isomeric forms of sugars and amino acids used for DNA and protein synthesis to cell polarization up to morphogenesis of the embryo [42–45], molecules, cells and organisms must be asymmetric. For the highly polarized cilia and flagella, chirality is also present at the level of the axoneme, and dynein arms invariably form a chiral pattern. Motile flagellated cells such as sperm must break the flagellar beat symmetry for navigation. Using theoretical arguments, we show that changing the swimming direction can be accomplished by beat patterns that break the space–time symmetry, either by a non-zero average flagellar curvature or even (*n* = 2*j*) beat harmonics. Sea urchin sperm represents an example, where steering is accomplished mainly by a non-zero average flagellar curvature. Human sperm, on the other side, steer by regulating both the average curvature and the second harmonic of their flagellar beat.

Most studies characterizing the beating of the sperm flagellum have addressed only the role of the average curvature in steering. Possibly, the development of faster video cameras will allow resolution of the second harmonic contribution in additional species. Our analytical theory implies that for freely swimming cells, a non-zero average curvature has a higher impact on the rotational velocity than a second harmonic. Previous studies using tethered sperm overestimated the role of the second harmonic owing to the experimental conditions used [41]. When tethered, our analytical theory shows that the second harmonic has about the same impact on the rotational velocity as a non-zero average curvature.

For navigation, sperm featuring a non-zero average curvature or a second harmonic must translate sensory information into a modulation of any of the three parameters *C*_0_, *C*_2_ or *ϕ*. The beat waveform of ciliated cells is regulated by the absolute intracellular Ca^2+^ concentration ([Ca^2+^]_i_) [46], as well as the temporal changes of this concentration (d[Ca^2+^]_i_/d*t* [47]). Receptors located on the sperm surface gather the sensory cues and initiate a signalling cascade. Downstream of this cascade, Ca^2+^ ions enter the cell via sperm-specific ion channels [9] or are released from intracellular stores [48]. For human sperm, it has been shown that the female hormone progesterone increases the [Ca^2+^]_i_ via CatSper channels and that stimulation with the female hormone progesterone also affects the second harmonic component [41]. For sea urchin sperm, a receptor guanylate cyclase captures species-specific chemoattractant peptides that are released by the egg. The resulting downstream Ca^2+^ influx via CatSper [49] and efflux via a Na^+^/Ca^2+^/K^+^ exchanger [50] result in a temporal modulation of [Ca^2+^]_i_. In these cells, the resulting swimming path curvature closely follows the time derivative d[Ca^2+^]_i_/d*t* of the calcium concentration [51]. From the functional dependence between rotational velocity and the average flagellar curvature, it can be inferred that *C*_0_ is controlled by the time derivative of [Ca^2+^], at least in sperm from marine invertebrates. This corollary, however, has not yet been tested experimentally.

The mechanism by which Ca^2+^ alters any of the steering parameters for directed cell movement is yet unclear. As a matter of fact, the molecular mechanism used by sperm to generate and change *C*_0_ and *C*_2_ is not known, yet some studies provide essential insights narrowing down the players leading to *C*_0_ generation. It has been proposed that the average curvature *C*_0_ can result from the controlled differential activity of molecular motors that produce the travelling wave. By increasing the activity of motors that bend the flagellum in one direction and/or reducing the activity of motors producing the opposite bending, an average curvature could be established. Such a mechanism is known as ‘biased switching’. An alternative mechanism consists of generating an average curvature by a separate set of molecular motor that does not contribute to the travelling wave. Such a mechanism, known as the ‘biased baseline’, has been favoured in quantitative studies of the flagellar waveforms from sea urchin sperm [31]. Along the same lines, recent studies characterizing the beating of isolated flagella from the green alga *Chlamydomonas* demonstrate that the average flagellar curvature can be generated in the absence of a travelling wave and *vice versa* [52], thus favouring the ‘biased baseline’ model. In addition, both the generation of average curvature and that of a travelling wave are active processes with different ATP requirements. The large amplitude of *C*_0_ in these flagella results in a highly asymmetric beating that resembles that from motile cilia in ciliary carpets, suggesting that a similar control of average curvature might be adopted by cilia and flagella.

The mechanisms and molecular players leading to a second harmonic generation and control are at present guesswork [41]. It has been suggested that second harmonic components result from a specialized function of the two dynein arms of the axoneme. This proposition is based on the different (by a factor of 2) beat frequencies observed for isolated axonemes with deficiencies on either dynein arm. Alternatively, elastic nonlinearities of the flagellum will result in second beat harmonics. Ca^2+^ binding to axonemal proteins might result in changes of the elastic nonlinearities. Sperm could exploit such a mechanism to alter the amplitude or phase of the second harmonic during navigation. Finally, it has been observed that activation of isolated axonemes from *Chlamydomonas* produces resonance frequencies with two peaks, at 30 and 60 Hz, indicating that second harmonics are also broadly present in nature [53,54].

In this review, we have considered prescribed prototypical flagellar beat patterns. However, the flagellum is elastic, and the waveform of the flagellar beat emerges by the interplay of active motor forces, elasticity and hydrodynamic drag forces. The resultant waveform compliance manifests itself in a load-response of the flagellar beat, i.e. speed and shape of flagellar bending waves depend on the hydrodynamic drag [55]. In previous work, where the flagellum was modelled as a simple elastic rod, bending due to the action of a homogeneous motor activity along the rod length was sufficient to explain the increasing bending amplitude towards the tip of the flagellum observed in experiments [41]. Elasticity also allows for an interplay of both mechanisms; for example, the torque generated by a second harmonic can result in an average curvature [41]. Similarly, drag induced by sperm swimming may deform [56] or buckle [57] the flagellum and further enhance the rotational velocity.

In the future, it would be interesting to determine which factors favour the use of second harmonics in human sperm and whether second harmonics of the beat play a role for the navigation of sperm from other species or ciliary beat in general.

## 7. Methods

### (a) Sperm motility

Single sperm cells were imaged using dark-field microscopy as previously described [41,47]. Images were collected at 500 frames per second using a high-speed CMOS camera (Dimax HD; PCO). The flagellar tracking of sea urchin sperm was done using a custom-made ImageJ plugin. In short, images were binarized using a high threshold (‘intermodes’ method). Thresholding resulted in isolated heads. The head centre and orientation angle were then calculated using the ImageJ particle analysis tool. The flagellar shape was obtained by applying a low threshold to the original image, and the flagellar coordinates were obtained by the ‘skeletonize’ operation. For human sperm, tracking was done as previously described [41]. In short, a custom-made software written in MATLAB (Mathworks) identified the best threshold for binarization of the image. Threshold increased until the resulting binary image had the expected cell area and coarse flagellar length. This was followed by a skeleton operation to identify the flagellum. The position of the head was determined by fitting an ellipse around the tethering point. The head orientation vector was defined as the vector parallel to the major axis of the ellipse and pointing towards the cell front. The orientation angle was defined as the angle between the orientation vector and the axis of the abscissa.

### (b) Flagellar analysis

The rotational velocity *Ω* was obtained from the time derivative of the head orientation angle. The mean curvature *C*_0_(*t*) was computed as the average of the flagellar curvature over the full arc-length. To estimate the power spectrum of the flagellum, the flagellar curvature was first smoothed by principal component analysis and the eigenmodes contributing less than 5% of the signal were discarded as noise. A Blackman–Harris window of size *l* = 250 ms for sea urchin and *l* = 500 ms for human sperm was used to determine time-dependent spectral quantities. The largest peak of the spectrum was associated with the fundamental beat frequency *ω*_0_(*t*) with amplitude *C*_1_. The second harmonic amplitude *C*_2_ is the integral over 2*ω*_0_ ± Δ*ω*_0_, with Δ*ω*_0_ = 1/*l*. A Gaussian filter of size 250 ms for sea urchin and 300 ms for human sperm was used to filter out the high-frequency oscillations in all temporal variations of parameters. Finally, the unknown phase shift *ϕ*_eff_ of the second harmonic contribution was determined by maximizing the correlation between *C*_2_sin*ϕ*_eff_ and *Ω*/*ω* using the correlation coefficient defined in [41]. All computations were implemented using Python.

### (c) Numerical computation of swimming velocities

We computed translational and rotational velocities of model sperm for the prototypical flagellar beat patterns of equations (3.4*a*–*c*) using resistive-force theory [25]. To facilitate comparison with the analytical theory, we likewise considered a headless sperm model. For the drag anisotropy ratio of the slender flagellum, we used the value *ξ*_R_ = *ξ*_⊥_/*ξ*_||_ = 1.81 [30]. Instantaneous velocities were computed at each instance in time by solving a linear system of equations comprising a force balance and a torque balance equation (electronic supplementary material, note S1) [58,59]. From the instantaneous translational and rotational velocities, we computed the resulting rotation of the material frame of the cell, as well as the swimming path. All computations were implemented using MATLAB (Mathworks).

The sperm flagellar tracking data, the software used for its analysis, and the software used for computing the rotational velocity can be found in the following repository: https://doi.org/10.5281/zenodo.3383174.

## Supplementary Material

Supplementary Material

## Supplementary Material

Supplementary Movie

## Data accessibility

The datasets and analysis scripts supporting this article are available on Zenodo: https://doi.org/10.5281/zenodo.3383174.
