# Supplementary material for: The steering gaits of sperm
Source: Philos Trans R Soc Lond B Biol Sci. 2019 Dec 30;375(1792):20190149. doi: 10.1098/rstb.2019.0149 (PMC7017342; doi:10.1098/rstb.2019.0149)
Supplement: Supplementary Material [file rstb20190149supp1.pdf]

Supplementary Information for the article
The steering gaits of sperm

A. Gong[1], S. Rode[2], U.B. Kaupp[1], G. Gompper[2], J. Elgeti[2], B. M. Friedrich[3], and L. Alvarez[1]

[1]Center of Advanced European Studies and Research (caesar), Molecular Sensory Systems, Ludwig-Erhard-Allee 2, 53175 Bonn, Germany.

[2]Theoretical Soft Matter and Biophysics, Institute of Complex Systems and Institute for Advanced Simulation, Forschungszentrum Jülich, 52425 Jülich, Germany.

[3]Biological Algorithms Group, TU Dresden, Biological Systems Path of the Center for Advancing Electronics Dresden (cfaed), Helmholtzstr. 18, 01069 Dresden Germany.

This Supplementary Information contains the following:

1- **Supplementary Note 1**: calculation in the small-curvature approximation of the sperm rotation velocity.

2- **Supplementary Note 2**: demonstration that the sum of a sinusoidal travelling wave and its second harmonic can be written as a sinusoidal travelling wave with a time-dependent amplitude and phase.

3- **Supplementary Note 3**: comparison of different means found in the literature of quantifying flagellar bending waves.

Supplementary Note 1

In the following, we describe a calculation of the rotation velocity in the small-curvature approximation regime for tethered and freely swimming sperm. The sperm models are assumed head-less for simplicity, and thus, any drag and torque on the head are neglected. Flagellar beats are assumed planar. In the limit of small Reynolds numbers, the freely swimming cell is free from external forces and torques, while the tethered cell, being still free to rotate around its head is assumed to be free from external torques.

We consider a beat waveform characterised by a flagellar curvature $C(s,t)$

$$\begin{aligned} C(s,t) &= C_0 + C_1 \cos(ks - \omega_0 t) \\ C(s,t) &= C_1 \cos(ks - \omega_0 t) + C_n \sin(ks - n\omega_0 t + \phi) \end{aligned} \quad \text{(S1.1).}$$

Here, C_1 and C_n are the first and the n-th harmonic curvature amplitude, respectively, ϕ is the phase difference between first and the n-th harmonic, and C_0 is the average curvature. To implement the small-curvature approximation, we introduce an arbitrary variable $\varepsilon << 1$ and variables C_{i*} (with i = 0, 1, 2, …, n) such that

$$\begin{aligned} C_0 &= \varepsilon C_{0*} \\ C_1 &= \varepsilon C_{1*} \\ C_n &= \varepsilon C_{n*} \end{aligned} \quad \text{(S1.2)}$$

By substituting equations (S1.2) into equations. (S1.1) we find

$$\begin{aligned} C(s,t) &= \varepsilon\left[C_{0*} + C_{1*} \cos(ks - \omega_0 t)\right] \\ C(s,t) &= \varepsilon\left[C_{1*} \cos(ks - \omega_0 t) + C_{n*} \sin(ks - n\omega_0 t + \phi)\right] \end{aligned} \quad \text{(S1.3)}$$

In the material frame of the cell (see Supplementary Note 3) with base vectors $\mathbf{e}_1(t)$ and $\mathbf{e}_2(t)$, the angle $\psi(s,t)$ between the flagellar tangent and the long axis of the sperm head ($\mathbf{e}_1$) is [1]

$$\psi(s,t) = \int_0^s \mathrm{d}s' C(s',t) + \psi(0,t).$$

Thus, at time t, the flagellar centerline coordinates $\mathbf{r}(s,t)$ in the material frame of the cell reads

$$\mathbf{r}(s,t) = \mathbf{r}(0,t) + \int_0^s \cos\psi(s',t)\,\mathrm{d}s'\mathbf{e_1} + \int_0^s \sin\psi(s',t)\,\mathrm{d}s'\mathbf{e_2}. \quad \text{(S1.4)}$$

The tangent vector $\mathbf{t}(s,t) = \partial_s \mathbf{r}(s,t) = \mathbf{e}_1 \cos\psi(s,t) + \mathbf{e}_2 \sin\psi(s,t)$ is normalized by virtue of equation (S1.4). We also introduce the local normal vector $\mathbf{n}(s,t) = -\mathbf{e}_1 \sin\psi(s,t) + \mathbf{e}_2 \cos\psi(s,t)$, which is always perpendicular to $\mathbf{t}(s,t)$.

We consider the hypothetical case of a sperm cell whose head is infinitesimally small and is constrained from both translating and rotating. Specifically, $\mathbf{r}(0,t)$ = (0,0) and the material frame vectors $\mathbf{e}_1$, $\mathbf{e}_2$ will be assumed constant. The velocity for each small segment along the flagellum depends only on active beating and is given by

$$\mathbf{v}(s,t) = \partial_t \mathbf{r}(s,t). \quad \text{(S1.5)}$$

The drag force density opposing to the active flagellar beat reads

$$\mathbf{f}_{\mathrm{a}}(s,t) = -\xi_{\parallel}(\mathbf{v}\cdot\mathbf{t})\mathbf{t} - \xi_{\perp}(\mathbf{v}\cdot\mathbf{n})\mathbf{n}\,. \tag{S1.6}$$

Thus, the active force and torque generated by the whole flagellum can be calculated as follows:

$$\begin{aligned} \mathbf{F}_{\mathrm{a}} &= \int_0^{\mathrm{L}} \mathbf{f}_{\mathrm{a}}(s,t)\mathrm{d}s \\ \mathbf{T}_{\mathrm{a}} &= \int_0^{\mathrm{L}} \mathbf{r}(s,t)\times\mathbf{f}_{\mathrm{a}}(s,t)\mathrm{d}s, \end{aligned} \tag{S1.7}$$

where *L* is the flagellar length. In fact, -$\mathbf{F}_{\mathrm{a}}$ and -$\mathbf{T}_{\mathrm{a}}$ are the constraining force and torque required to restrain the sperm head from moving, while the sperm flagellum is beating. As discussed in the main text, for a freely swimming cell, the active force $\mathbf{F}_{\mathrm{a}}$ and torque $\mathbf{T}_{\mathrm{a}}$ that result from beating will be instantaneously balanced by the drag force $\mathbf{F}_{\mathrm{r}}$ and torque $\mathbf{T}_{\mathrm{r}}$ that result from the global translation and rotation, i.e., from the rigid body motion. Let us denote the instantaneous translational and rotational velocities of the material frame of the head of a freely swimming sperm cell as $\mathbf{v}_{\mathrm{H}}$ and Ω_{H}, respectively. Similar to equation (S1.6), we compute the drag force density resulting from the rigid body motion as

$$\mathbf{f}_{\mathrm{r}}(s,t) = -\xi_{\parallel}\left[(\mathbf{v}_{\mathrm{H}} + \boldsymbol{\Omega}_{\mathrm{H}}\times\mathbf{r})\cdot\mathbf{t}\right]\mathbf{t} - \xi_{\perp}\left[(\mathbf{v}_{\mathrm{H}} + \boldsymbol{\Omega}_{\mathrm{H}}\times\mathbf{r})\cdot\mathbf{n}\right]\mathbf{n}\,, \tag{S1.8}$$

where $\boldsymbol{\Omega}_{\mathrm{H}} = \Omega_{\mathrm{H}}\mathbf{e}_1\times\mathbf{e}_2$. The net drag force and torque are thus

$$\begin{aligned} \mathbf{F}_{\mathrm{r}} &= \int_0^{\mathrm{L}} \mathbf{f}_{\mathrm{r}}(s,t)\mathrm{d}s \\ \mathbf{T}_{\mathrm{r}} &= \int_0^{\mathrm{L}} \mathbf{r}(s,t)\times\mathbf{f}_{\mathrm{r}}(s,t)\mathrm{d}s. \end{aligned} \tag{S1.9}$$

The instantaneous translational ($\mathbf{v}_{\mathrm{H}}$) and rotational (Ω_{H}) velocities of the material frame (seen from the lab frame) can be calculated from the equation set resulting from the torque-free and force-free conditions:

$$\begin{cases} \mathbf{F}_{\mathrm{a}} + \mathbf{F}_{\mathrm{r}} = 0 \\ \mathbf{T}_{\mathrm{a}} + \mathbf{T}_{\mathrm{r}} = 0 \end{cases} \quad \text{(S1.10).}$$

Finally, the mean rotational velocity can be derived from the as-calculated instantaneous variables:

$$\Omega = <\Omega_H> = \frac{\omega_0}{2\pi} \int_0^{2\pi/\omega_0} \Omega_H \mathrm{d}t \qquad \text{(S1.11).}$$

Here, $2\pi/\omega_0$ indicates the beat period. For a tethered sperm cell that is free to rotate around the position of its head, the net torque vanishes, while net active force is equal in magnitude and opposite to the constraining force applied at the tethering point. Thus, only the torque balance has to be considered. By construction, $\mathbf{v}_H = (0, 0)$.

We used the symbolic toolbox of MATLAB to obtain analytical solutions of equation (S1.10) for different harmonic contributions, retaining only the leading terms in an expansion in a small-curvature approximation parameter ε (see equation S1.3). The results for freely swimming and tethered sperm are summarised in Supplementary Tables 1 and 2, respectively, for the special case $k = 2\pi/L$. For $n = 4$, only the approximate expression for Ω is provided. The complete solution for this or other harmonics can be calculated using the corresponding MATLAB code online: https://doi.org/10.5281/zenodo.3383174.

Condition	Analytical solution for Ω	Approximation*
C_0 & C_1	$-\frac{\varepsilon^3 \omega_0 C_{1*}^2 C_{0*}}{k^3} \cdot \frac{(36-\pi^4)+(9\pi^2-72)\xi_R^{-1}+(\pi^4\text{-}3\pi^2)\xi_R}{2\pi^4} + O(\varepsilon^4)$	$-0.36 \cdot \frac{\omega_0 C_1^2 C_0}{k^3}$
C_1	0	0
C_2 & C_1	$-\frac{\varepsilon^3 \omega_0 C_{1*}^2 C_{2*} \sin\phi}{k^3} \cdot \frac{9[(21\text{-}5\pi^2)+(3\pi^2\text{-}10)\xi_R^{-1}+(2\pi^2\text{-}8)\xi_R]}{8\pi^4} + O(\varepsilon^4)$	$-0.043 \cdot \frac{\omega_0 C_1^2 C_2 \sin\phi}{k^3}$
C_3 & C_1	0	0
C_4 & C_1	$\Omega(C_1^4, C_4) + O(\varepsilon^6)$	$0.015 \cdot \frac{\omega_0 C_1^4 C_4 \sin\phi}{k^5}$

Supplementary Table 1. Calculated rotation velocity for the small-curvature approximation for freely swimming sperm with different harmonic contributions. *Only the leading term in ε is used and the drag coefficient ratio is assumed to be $\xi_R = 1.81$. To obtain the final expression, equations (S1.2) have been used.

Condition	Analytical solution for Ω	Approximation*
**C_0 & C_1**	$-\frac{\varepsilon^3\omega_0 C_{1*}^{\ 2} C_{0*}\left(2\pi^2-3\right)}{8\pi^4 k^3}\left[\left(\pi^2-3\right)-\left(\pi^2-6\right)\xi_R^{-1}\right]+O(\varepsilon^4)$	$-0.102\frac{\omega_0 C_1^{\ 2} C_0}{k^3}$
**C_1**	0	0
**C_2 & C_1**	$\frac{\varepsilon^3 3\omega_0 C_{1*}^{\ 2} C_{2*}}{2^7\pi^4 k^3}[\left(33-10\pi^2-66\xi_R^{\ -1}+8\pi^2\xi_R^{\ -1}\right)\sin\phi$ $+\left(24\pi^3+9\pi-24\pi^3\xi_R^{\ -1}-18\pi\xi_R^{\ -1}\right)\cos\phi]+O(\varepsilon^4)$	$0.081\cdot\frac{\omega_0 C_1^{\ 2} C_2\sin\phi_{eff}}{k^3}$, $\phi_{eff}\approx\phi+0.56\pi$
**C_3 & C_1**	0	0
**C_4 & C_1**	$\Omega(C_1^{\ 4},C_4)+O(\varepsilon^6)$	$0.011\cdot\frac{\omega_0 C_1^{\ 4} C_4\sin\phi_{eff}}{k^5}$, $\phi_{eff}\approx\phi-0.15\pi$

Supplementary Table 2. Calculated rotation velocity for the small-curvature approximation for tethered sperm with different harmonic contributions. *Only the leading term in ε is used and the drag coefficient ratio is assumed to be ξ_R = 1.81. To obtain the final expression, equations (S1.2) have been used.

Supplementary Note 2

In the following, we demonstrate that the sum of a symmetric sinusoidal travelling wave and its second harmonic can be written as a sinusoidal symmetric travelling wave with a time-dependent amplitude and phase. Departing from equation (3.4c) of the main text

$$C(s,t)=C_1\sin(ks-\omega_0 t)+C_2\sin(ks-2\omega_0 t+\phi). \qquad \text{(S2.1)}$$

Equation (S2.1) can be rewritten using Euler's formula as

$$C(s,t)=\mathrm{Im}\left\{C_1 e^{\mathrm{i}(ks-\omega_0 t)}+C_2 e^{\mathrm{i}(ks-2\omega_0 t+\phi)}\right\}=\mathrm{Im}\left\{e^{\mathrm{i}(ks-\omega_0 t)}(C_1+C_2 e^{\mathrm{i}\beta})\right\}, \qquad \text{(S2.2)}$$

where Im is the imaginary part operator and $\beta = -\omega_0 t + \phi$ can be seen as a time-dependent phase between the first and second harmonic components. This phase can be generalised for any sum of harmonics, but for the sake of simplicity, we will adhere to the second harmonic case.

Using Euler's formula and the polar representation of a complex number, we find

$$C_1+C_2 e^{\mathrm{i}\beta}=\sqrt{C_1^2+2C_1C_2\cos\beta+C_2^2}\cdot e^{\mathrm{i}\tan^{-1}\left(\frac{C_2\sin\beta}{C_1+C_2\cos\beta}\right)}. \qquad \text{(S2.3)}$$

Substituting equation (S2.3) into equation (S2.2), it is readily found

$$C(s,t)=\sqrt{C_1^2+2C_1C_2\cos\beta+C_2^2}\cdot\mathrm{Im}\left\{e^{\mathrm{i}\left[ks-\omega_0 t+\tan^{-1}\left(\frac{C_2\sin\beta}{C_1+C_2\cos\beta}\right)\right]}\right\}. \qquad \text{(S2.4)}$$

Using Euler's formula again and applying the imaginary part operator, the anticipated expression can be found

$$C(s,t)=\overline{C}\sin\left(ks-\omega_0 t+\Phi\right), \qquad \text{(S2.5)}$$

where $\Phi(t)$ and $\overline{C}(t)$ denote time-dependent phase and amplitude given by:

$$\begin{aligned}\Phi(t)&=\tan^{-1}\left(\frac{C_2\sin(\phi-\omega_0 t)}{C_1+C_2\cos(\phi-\omega_0 t)}\right)\\ \overline{C}(t)&=\sqrt{C_1^2+2C_1C_2\cos(\phi-\omega_0 t)+C_2^2}\end{aligned} \qquad \text{(S2.6).}$$

In summary, the superposition of a sinusoidal travelling wave and its second harmonic (equation S2.1) can be written as a single sinusoidal travelling wave (equation S2.5). This function is symmetric in space at any point in time, and its time average is zero. Yet, it features a time-dependent amplitude and phase that results in an asymmetric beat envelope.

Supplementary Note 3

This note compares different means found in the literature of characterising in quantitative terms the flagellar bending waves. We restrict ourselves to planar beat patterns.

In this study, we quantify the flagellar beat in terms of the *curvature* $C(s,t)$ of the flagellar centerline as a function of arc length s and time t. We employ a signed version of curvature, where positive ($C(s,t)>0$) and negative ($C(s,t)<0$) values indicate a bend in a counter-clockwise and clockwise direction, respectively (when moving along the flagellar centerline from its proximal towards its distal end). Geometrically, the inverse of the curvature defines the local radius of curvature, $r = 1/|C(s,t)|$(Supplementary Fig. 1). The characterisation in terms of curvature $C(s,t)$ is independent of the choice of the coordinate system and allows to uniquely reconstruct the flagellar shape at any time t (up to any translation or rotation in the plane).

In the last half a century, alternative quantifications have been used to characterise flagellar bending waves: *tangent angle* [1-3], the *shear angle* [4-6], and the *bend angles* [7, 8]. We review these below and discuss their equivalence to the representation in terms of curvature.

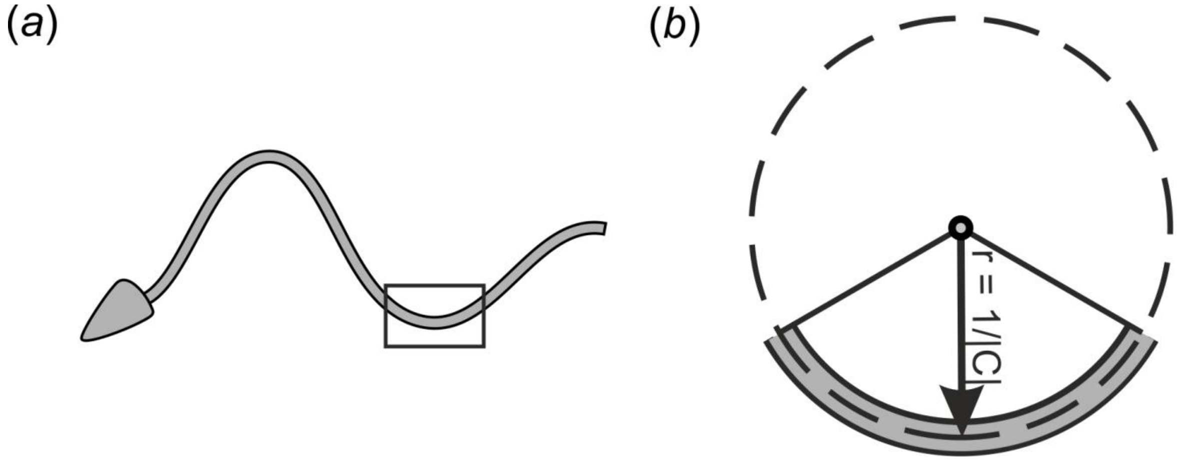

Supplementary Figure 1. Definition of the flagellar curvature. (*a*) sketch of a sperm cell. (*b*) Magnified flagellar segment corresponding to that segment boxed in (*a*). The radius of curvature equals the radius of the local coalescence circle that snugly fits the flagellar centerline at arc length position *s*. The flagellar curvature is defined as the inverse of this radius.

Tangent angle ψ representation

For the tangent angle representation, a material frame with base vectors $\mathbf{e}_1$ and $\mathbf{e}_2$ is introduced (Supplementary Fig. 2). A common choice defines the base vectors in terms of the long and the short axis of an approximately spheroidal sperm head. The tangent angle $\psi(s,t)$ is then defined as the angle between the tangent to the flagellar centerline and the $\mathbf{e}_1$-direction. It is straightforward to convert this tangent angle representation to the curvature representation using the geometrical definition of signed curvature as the derivative of the tangent angle with respect to arc length *s*

$$C(s,t) = \partial_s \psi(s,t) \,. \qquad \text{(S3.1)}$$

For the sake of explicitness, we consider an idealised beat pattern with tangent angle

$$\psi(s,t) = A_0 s + A_1 \cos(ks - \omega_0 t) \,, \qquad \text{(S3.2),}$$

where *k* is the wavenumber (i.e., spatial frequency) and ω_0 denotes the angular beat frequency. We readily find:

$$C(s,t) = A_0 - A_1 k \sin(ks - \omega_0 t) \,. \qquad \text{(S3.3)}$$

By comparing equation (S3.3) with equation (3.4b) in the main text, we see that $C_0 = A_0$ and $C_1 = -A_1k$.

A practical advantage of the tangent angle is that its estimation is less sensitive to measurement noise as compared to the curvature $C(s,t)$. Flagellar curvature $C(s,t)$ is sensitive to measurements errors because uncertainties of flagellar tracking become amplified by taking the spatial derivative (see equation S3.1).

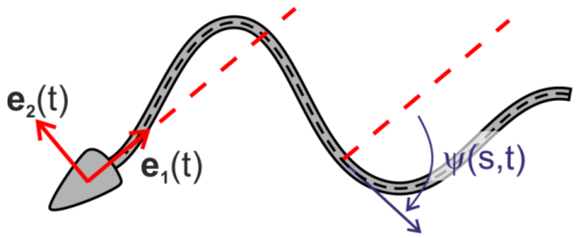

Supplementary Figure 2. Definition of tangent angle. The material frame of the cell is defined by the two orthonormal vectors $\mathbf{e}_1(t)$ and $\mathbf{e}_2(t)$, parallel and perpendicular to the long axis of the sperm head, respectively. Dashed black line represents the flagellar centerline. The tangent angle $\psi(s,t)$ is the angle between the tangent to the flagellum at arc length position *s* and time *t* and the direction of material frame vector $\mathbf{e}_1(t)$.

Shear angle θ_{shear} representation

The shear angle is also extensively used for describing the flagellar bending [4-6]. It is defined as the tangent angle at an arc length point *s* minus the tangent angle at the flagellar base (Supplementary Fig. 3):

$$\theta_{\mathrm{shear}}(s,t) = \psi(s,t) - \psi(0,t)\,. \qquad \text{(S3.4)}$$

For the special case $\psi(0,t) = 0$, i.e., where the proximal end of the flagellum is parallel to the vector $\mathbf{e}_1$ of the material frame, shear angle and tangent angle are identical. The shear angle representation (Supplementary Fig. 3*a*-*b*) was exploited by C.J. Brokaw to explain flagellar bend propagation based on a model of sliding microtubules [6]. This parameter was generally used as a proxy for the shear displacement between axonemal tubules [9, 10] based on the assumption that at the basal end of the axoneme, microtubules have no relative displacement (Supplementary Fig. 3*c*-*d*). Note, however, that previous studies [11-13] have challenged this assumption.

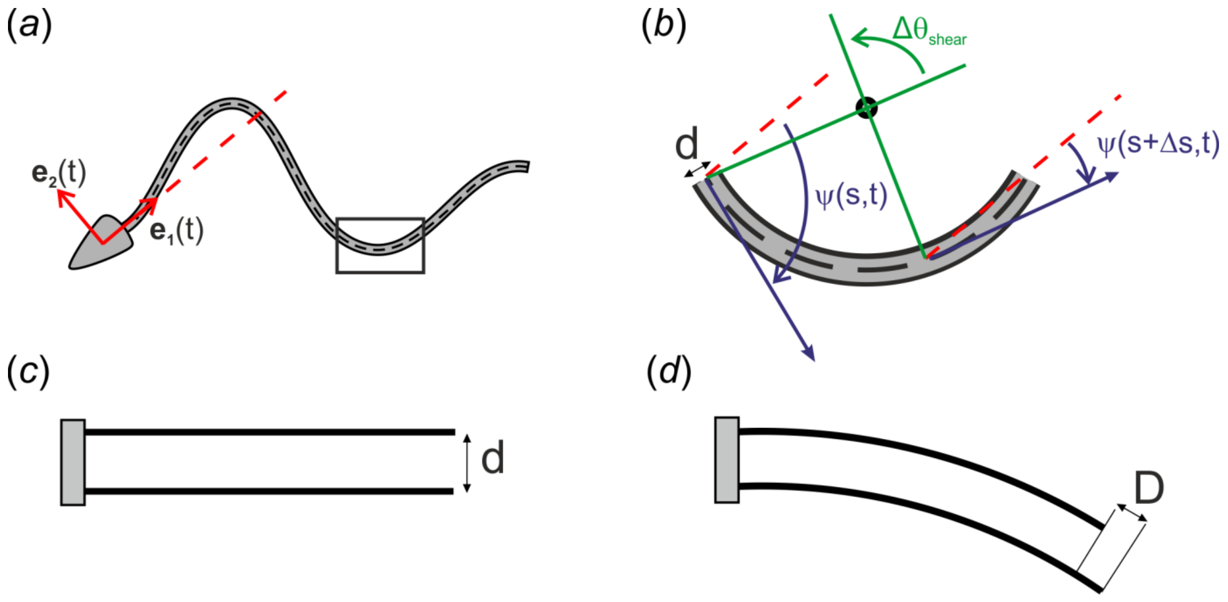

Supplementary Figure 3. Shear displacement description. (*a*) Sperm cell with its corresponding reference frame (red arrows) and the flagellar centerline (black dashed line). (*b*) Magnified flagellar portion with cross-section diameter *d* corresponding to the boxed segment shown in (*a*). The small shear angle $\Delta\theta_{shear}$ represents the angle subtended by the arc between arc length coordinates *s* and *s* + Δ*s*. (*c-d*) Sketch of two microtubule doublets clamped at the base before (*c*) and after (*d*) bending. Internal flagellar forces result in bending and bending results into a sliding shear displacement *D* between microtubules.

The shear angle is closely related to the local shear displacement between opposing microtubules. From Supplementary Fig. 3 we can appreciate that $\Delta\theta_{shear} = \psi(s+\Delta s,t) - \psi(s,t)$. For $\Delta s \to 0$, we have:

$$\mathrm{d}\theta_{\text{shear}} = \partial_s \psi(s,t)\mathrm{d}s = C(s,t)\mathrm{d}s\,. \qquad \text{(S3.5)}$$

The shear displacement between opposing doublets within this infinitesimal region is:

$$\mathrm{d}D_{\text{shear}} = \left(r + d/2\right)\mathrm{d}\theta_{\text{shear}} - (r - d/2)\mathrm{d}\theta_{\text{shear}} = d\cdot\mathrm{d}\theta_{\text{shear}}\,. \qquad \text{(S3.6)},$$

where *d* is the axonemal diameter and *r* is the radius of curvature at position *s*. Thus, the total shear displacement at position *s* is given by:

$$D_{\text{shear}} = \int_0^s \mathrm{d}D_{\text{shear}} = d\int_0^s \mathrm{d}\theta_{\text{shear}} = d[\psi(s,t) - \psi(0,t)] = d\cdot\theta_{\text{shear}}(s,t)\,. \qquad \text{(S3.7)}$$

The tangent angle and the shear angle representations only differ by a (possibly time-dependent) constant. Analogous to equation (S3.1), the derivative of the shear angle with respect to arc length *s* yields again the curvature.

Bend angles θ_P and θ_R representation

Previous authors used yet a fourth quantification of flagellar bending waves in terms of bend angles (θ_P and θ_R)[7, 8]. For this representation, the flagellar shape is approximated as a sequence of alternating bends forming circular arcs connected by straight segments [8, 14]. Bends are termed principal (reverse) bends when their convex side faces outwards (inwards) from the swimming path (Supplementary Fig. 4).

For a flagellar shape that is approximately sinusoidal, the arc of a reverse bend meets the arc of a principal bend at a so-called inflexion point. The angles between the tangent directions of two such inflexion points define the bend angles, θ_P and θ_R, respectively (Supplementary Fig. 4). Bend angles θ_P and θ_R have opposite signs, and the absolute value of θ_P is larger (or at least equal) to that of θ_R, $|\theta_P| \geq |\theta_R|$. Note that previous studies used the symbols θ_P and θ_R for the respective absolute values. For clarity, we will explicitly express the absolute value using the modulus $|\theta_P|$ and $|\theta_R|$. The difference between the absolute values, $\Delta\theta = |\theta_P| - |\theta_R|$ provides a measure for the asymmetry of the flagellar beat.

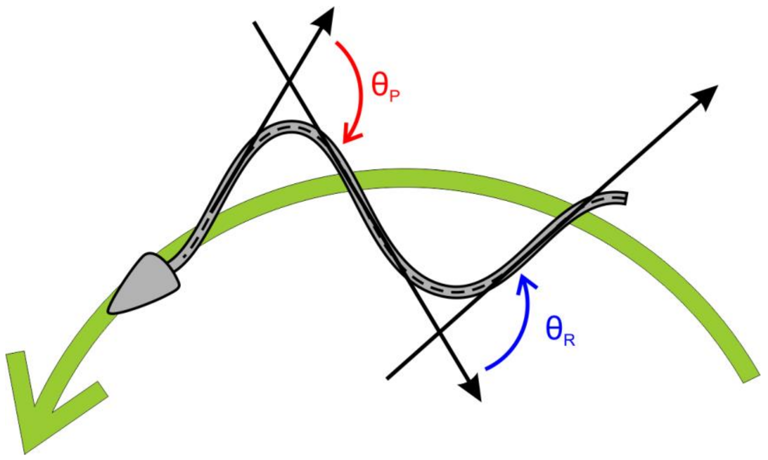

Supplementary Figure 4. Definition of bend angles θ_P and θ_R. The flagellar waveform is characterised by bending arcs in opposite directions interspaced by straight regions. The principal (reverse) bends have their convex side faces outwards (inwards) from the swimming path (green arrow). The corresponding principal and reverse bend angles are referred to as θ_P (red) and θ_R (blue).

Unlike for the tangent $\psi(s,t)$ or the shear angle $\theta_{shear}(s,t)$ representations, the flagellar waveform cannot be fully reconstructed from the two values of the bend angles θ_P and θ_R. However, we can derive a conversion between both the bend angle representation and the curvature representation for an idealised flagellar beat pattern, such as the one given by equation (S3.3). The "connection points" between the principal and the reverse arcs are characterised by zero curvature

$$C(s,t) = \partial_s \psi(s,t) = 0 .$$

For the idealised flagellar beat pattern from equation (S3.3), these inflexion points are located at arc length positions for integer n

$$s_n = \frac{1}{k}[n\pi + (-1)^n \arcsin(\frac{A_0}{A_1 k}) + \omega_0 t] \qquad (n = 0,1,2\cdots) \tag{S3.8}$$

Substituting equation (S3.8) into equation (S3.2), we obtain the corresponding value of the tangent angle ψ. The resulting signed bend angles correspond to the case for $|\theta_P| > |\theta_R|$. Thus

$$\begin{aligned} \theta_P &= \Psi(s_{2n},t) - \Psi(s_{2n-1},t) = \frac{A_0}{k}[\pi + 2\arcsin(\frac{A_0}{A_1 k})] + 2A_1 \cos(\arcsin(\frac{A_0}{A_1 k})) \\ \theta_R &= \Psi(s_{2n+1},t) - \Psi(s_{2n},t) = \frac{A_0}{k}[\pi - 2\arcsin(\frac{A_0}{A_1 k})] - 2A_1 \cos(\arcsin(\frac{A_0}{A_1 k})) \end{aligned} \tag{S3.9}$$

Using the identity $\cos(\arcsin(\frac{A_0}{A_1 k})) = \sqrt{1-(\frac{A_0}{A_1 k})^2}$, equation (S3.9) can be simplified to:

$$\theta_P = \frac{A_0}{k}[\pi + 2\arcsin(\frac{A_0}{A_1 k})] + 2A_1\sqrt{1-(\frac{A_0}{A_1 k})^2} \tag{S3.10}$$

$$\theta_R = \frac{A_0}{k}[\pi - 2\arcsin(\frac{A_0}{A_1 k})] - 2A_1\sqrt{1-(\frac{A_0}{A_1 k})^2} \tag{S3.11}$$

As previously shown, $C_0 = A_0$ and $C_1 = -A_1 k$. Equations. (S3.10-11) can be used to compute the asymmetry score $\Delta\theta$. For regular flagellar shapes (θ_R<0):

$$\Delta\theta = |\theta_P| - |\theta_R| = \frac{2\pi C_0}{k} .$$

This result shows that for simple flagellar beat patterns, the asymmetry score is proportional to the mean curvature C_0. Note that in the above derivation, we assumed C_0>0. For C_0<0, the expressions for θ_P and θ_R are swapped.

References

1. Friedrich, B.M., Riedel-Kruse, I.H., Howard, J., and Jülicher, F. (2010). High-precision tracking of sperm swimming fine structure provides strong test of resistive force theory. J. Exp. Biol. *213*, 1226-1234.
2. Wennemuth, G., Carlson, A.E., Harper, A.J., and Babcock, D.F. (2003). Bicarbonate actions on flagellar and Ca^{2+}-channel responses: initial events in sperm activation. Development *130*, 1317-1326.
3. Chattopadhyay, A.K., Hilfinger, A., and Jülicher, F. (2009). Nonlinear dynamics of cilia and flagella. Physical Review E *79*, 051918.
4. Brokaw, C.J. (1991). Microtubule sliding in swimming sperm flagella: direct and indirect measurements on sea urchin and tunicate spermatozoa. The Journal of cell biology *114*, 1201-1215.
5. Pelle, D.W., Brokaw, C.J., Lesich, K.A., and Lindemann, C.B. (2009). Mechanical properties of the passive sea urchin sperm flagellum. Cell motility and the cytoskeleton *66*, 721-735.
6. Brokaw, C.J. (1971). Bend propagation by a sliding filament model for flagella. The Journal of experimental biology *55*, 289-304.
7. Brokaw, C. (1979). Calcium-induced asymmetrical beating of triton-demembranated sea urchin sperm flagella. The Journal of Cell Biology *82*, 401-411.
8. Goldstein, S.F. (1977). Asymmetric waveforms in echinoderm sperm flagella. Journal of Experimental Biology *71*, 157-170.
9. Takei, G.L., Fujinoki, M., Yoshida, K., and Ishijima, S. (2017). Regulatory mechanisms of sperm flagellar motility by metachronal and synchronous sliding of doublet microtubules. MHR: Basic science of reproductive medicine *23*, 817-826.
10. Brokaw, C.J. (1989). Direct measurements of sliding between outer doublet microtubules in swimming sperm flagella. Science *243*, 1593-1596.
11. Vernon, G.G., and Woolley, D.M. (2002). Microtubule displacements at the tips of living flagella. Cell motility and the cytoskeleton *52*, 151-160.
12. Vernon, G.G., and Woolley, D.M. (2004). Basal sliding and the mechanics of oscillation in a mammalian sperm flagellum. Biophysical journal *87*, 3934-3944.
13. Riedel-Kruse, I.H., Hilfinger, A., Howard, J., and Jülicher, F. (2007). How molecular motors shape the flagellar beat. HFSP journal *1*, 192-208.
14. Brokaw, C.J. (1965). Non-sinusoidal bending waves of sperm flagella. The Journal of experimental biology *43*, 155-169.
